# Insect antimicrobial peptides: potential tools for the prevention of skin cancer

**DOI:** 10.1007/s00253-016-7718-y

**Published:** 2016-07-15

**Authors:** Miray Tonk, Andreas Vilcinskas, Mohammad Rahnamaeian

**Affiliations:** 1 LOEWE Center for Insect Biotechnology and Bioresources, Fraunhofer Institute for Molecular Biology and Applied Ecology, Winchester Strasse, 35394 Giessen, Germany; 2 Institute for Insect Biotechnology, Justus Liebig University of Giessen, Heinrich-Buff-Ring 26-32, 35392 Giessen, Germany

**Keywords:** Antimicrobial peptides, Prophylaxis, Skin cancer, Cosmetic industry, Sun cream

## Abstract

Antimicrobial peptides/proteins (AMPs) are biologically active molecules with diverse structural properties that are produced by mammals, plants, insects, ticks, and microorganisms. They have a range of antibacterial, antifungal, antiviral, and even anticancer activities, and their biological properties could therefore be exploited for therapeutic and prophylactic applications. Cancer and cancer drug resistance are significant current health challenges, so the development of innovative cancer drugs with minimal toxicity toward normal cells and novel modes of action that can evade resistance may provide a new direction for anticancer therapy. The skin is the first line of defense against heat, sunlight, injury, and infection, and skin cancer is thus the most common type of cancer. The skin that has been exposed to sunlight is particularly susceptible, but lesions can occur anywhere on the body. Skin cancer awareness and self-efficacy are necessary to improve sun protection behavior, but more effective preventative approaches are also required. AMPs may offer a new prophylactic approach against skin cancer. In this mini review, we draw attention to the potential use of insect AMPs for the prevention and treatment of skin cancer.

## Introduction

Despite efforts to develop effective new treatments, cancer remains the second most common cause of death in the world (Siegel et al. 2014). The worldwide occurrence of cancer is increasing, and in the case of skin cancer, one relevant factor is the prolonged exposure of the skin to the sun during normal daily activities or as a leisure pursuit, resulting in high doses of ultraviolet (UV) radiation (Holm 2014; Palanki et al. 2015). UV radiation from artificial sources, such as tanning beds and sunlamps, provides additional exposure scenarios (Palanki et al. 2015). General risk factors for skin cancer include lack of skin pigmentation, lesions such as moles, and compromised immunity caused by immunosuppressive medication (e.g., for organ transplants) or the presence of HIV/AIDS (Cakir et al. 2012; Chiao and Krown 2003; Eide et al. 2013; Iannacone et al. 2015; Zhu et al. 2015).

Approximately 3.5 million people are diagnosed with skin cancer annually in the USA (Robinson 2005; Rogers et al. 2010). There are three major types of skin cancer: basal cell carcinoma, squamous cell carcinoma, and malignant melanoma (Stern 2010). Basal cell carcinoma is the most common type, but it is also the least dangerous if detected early. It involves cells from the deeper layers of skin, usually in parts of the body that are exposed to the sun, such as the face, head, neck, ears, shoulders, and back, with most cases occurring on the face (Gordon and Carucci 2013; Telfer et al. 2008). Squamous cell carcinoma is the second most common type, involving cells of the upper skin layers, and is more likely to spread to areas under the skin. It commonly occurs on the legs and feet but may develop elsewhere (Ogden and Telfer 2009). Melanoma is the least common cancer of the skin but is among the most aggressive of known cancers (Vera et al. 2015). It can occur anywhere on the skin but is more likely to develop on the back or legs, and although curable in its early stages, the later-stage tumors often become drug resistant and therefore recalcitrant to chemotherapy (Korotkov and Garcia 2012; Ogden and Telfer 2009; Riedl et al. 2011a). Exposure to sun increases the risk of all three main types of skin cancer (Gallagher et al. 2010), but exposure during childhood is particularly harmful in the case of melanomas and basal cell carcinomas (Stewart and Wild 2014).

Skin cancer therapy involves the careful selection of one or more modalities, including surgery, radiotherapy and chemotherapy. Current drugs for such indications are insufficiently selective, resulting in deleterious effects against non-target cells, particularly those that normally divide rapidly (Al-Benna et al. 2011; Kalyanaraman et al. 2002), resulting in symptoms such as alopecia, rashes, vomiting, and even myelosuppression (Ferguson and Pearson 1996; Harris et al. 2013; Sanderson et al. 1996). Many tumors ultimately become resistant to conventional chemotherapy due to the selection of multidrug-resistant cells (Zahreddine and Borden 2013). It is therefore imperative to find new products with novel modes of action, such as cytotoxic antimicrobial peptides and proteins (AMPs) (Chamorro et al. 2009; Koczulla and Bals 2003).

AMPs have been developed as alternatives for the treatment of infectious diseases. Such peptides are produced naturally by nearly all organisms as a part of the innate immune system (Rahnamaeian et al. 2016; Tonk et al. 2014), and due to their broad antimicrobial spectrum, they are often described as natural antibiotics (Bolouri Moghaddam et al. 2015). Several insect AMPs also show cytotoxic effects against diverse cancer cell lines, such as mouse myeloma, melanoma, lymphoma, leukemia, breast cancer, and lung cancer (Iwasaki et al. 2009; Kang et al. 2012; Kim et al. 2013; Xiao et al. 2006). These anticancer peptides (ACPs) are characterized by high therapeutic efficacy, a low probability of resistance emerging in target cells, and limited or no toxicity against mammalian erythrocytes, macrophages, and fibroblasts (Barbault et al. 2003; Saido-Sakanaka et al. 2004; Yamada et al. 2005). ACPs are also easy to synthesize and modify, they penetrate tumors efficiently, and they are biocompatible (Borghouts et al. 2005; Thayer 2011). On the other hand, they are also immunogenic and susceptible to peptidase activity and clearance through the kidneys, reducing their effective therapeutic half-life in vivo and making them more appropriate for topical applications (McGregor 2008; Rahnamaeian and Vilcinskas 2015; Wiesner and Vilcinskas 2010).

Although the activity of insect ACPs against skin cancer has not been studied in detail, proof-of-principle studies involving the topical application of ACPs from other sources have been successful. Rodrigues et al. (2008) showed that gomesin from hemocytes of the spider *Acanthoscurria gomesian* can arrest the growth of murine melanoma B16F10-Nex2 cells when administered topically in a cream-based formulation. Also, Gerashchenko et al. (2014) showed that human β-defensin 2 (hBD-2) can inhibit the growth of human carcinoma cells by suppressing the expression of B-Raf, cyclin D1, and cyclin E, inducing the expression of p21^WAF1^ and activating pRB. The greater abundance of negatively charged membrane components such as sialic acid, phosphatidylserine and heparan sulfate makes cancer cells attract certain cationic amphipathic peptides (Wang et al. 2016; Riedl et al. 2011b). Particularly, phosphatidylserine in cancer cell membranes is targeted by temporin-1CEa, an AMP from the Chinese brown frog *Rana chensinensis* (Wang et al. 2016). Temporin-1CEa induces cell death in breast cancer cells by releasing pro-apoptotic factors from the mitochondria and also disrupts the plasma membrane by exposing phosphatidylserine, increasing plasma membrane permeability, and inducing membrane depolarization (Wang et al. 2013).

The active motifs of ACPs are short, so large-scale synthesis is cost-effective. Certain ACPs not only show intrinsic anticancer activity but also enhance the potency of conventional drugs (Gaspar et al. 2013; Hancock et al. 2006; Silva et al. 2012). There are currently 196 entries in the Antimicrobial Peptide Database (APD) (http://aps.unmc.edu/AP/database/antiC.php) describing peptides with anticancer activity. Most ACPs achieve cell membrane disruption by lytic activity or induce apoptosis in cancer cells through mitochondrial damage, in many cases leaving normal mammalian cells unharmed (Coffelt and Scandurro 2008; Hilchie et al. 2011). This review discusses the targets and active mechanisms of ACPs and highlights their potential as both prophylactic and therapeutic reagents indicated for the prevention and treatment of cancer. We also consider the potential inclusion of ACPs in cosmetics and personal care products, especially sun protection creams that could enhance protection against skin cancer by eliminating nascent cancer cells before symptoms become evident.

## The structure of ACPs

Insect AMPs are cationic and amphipathic, and although the length, sequence and structure may vary, most have a comparatively low molecular mass (≥10 kDa). The structure includes hydrophilic and hydrophobic regions, and the net charge is highly positive (Dennison et al. 2006). The structure of AMPs allows strong electrostatic binding with bacterial or fungal cell membranes and certain enveloped viruses (Hoskin and Ramamoorthy 2008; Reddy et al. 2004), but ACPs also have the unique ability to bind cancer cell membranes. Most ACPs contain six cysteine residues forming three intramolecular disulfide bonds that assemble into hairpin like α-helices, β-sheets, or mixed structures, but some extended structures have also been reported (Bulet and Stocklin 2005; Hoskin and Ramamoorthy 2008; Wang et al. 2013).

## The activity of ACPs

AMPs can be assigned to different classes according to their diverse physicochemical properties, but only two general modes of action have been described: membranolytic and non-membranolytic (Schweizer 2009). The activity of ACPs depends on their physicochemical characteristics, such as the primary sequence, secondary structure, net electric charge, amphipathicity, hydrophobicity, and concentration, as well as the composition of the target membrane (Adams et al. 2009; Reddy et al. 2004; Teixeira et al. 2012). The ability of many AMPs to permeabilize cell membranes correlates with their antimicrobial activities, e.g., in the case of defensins and cecropins (Rahnamaeian 2011). Membrane disruption by AMPs may involve pore formation (barrel-stave and toroidal pore models), membrane thinning, membrane dissolution (carpet-like model), or lipid-peptide domain formation. In other cases, AMPs bind to intracellular targets in the pathogen including nucleic acids and proteins (Bechinger and Lohner 2006; Brogden 2005; Chan et al. 2006; Papo and Shai 2005; Rahnamaeian et al. 2015; Yeaman and Yount 2003). Certain AMPs also display immunomodulatory activities (Jerala and Porro 2004; McPhee et al. 2005) such as the stimulation of chemokine and cytokine production and leukocyte chemotaxis (Bowdish et al. 2005). The ability of ACPs to kill tumor cells is poorly understood, although both membranolytic and non-membranolytic mechanisms may be involved. For example, several AMPs that interact with and disrupt negatively charged bacterial membranes (Hancock and Chapple 1999; Merrifield et al. 1995) can also kill mammalian cancer cells by inducing membrane permeability or apoptosis (Iwasaki et al. 2009; Papo and Shai 2005). ACPs are also able to lyse the tumor cells by inducing the blebbing and permeabilization of the membrane after binding directly to plasma membrane phospholipids such as phosphatidylinositol 4,5-bisphosphate (PIP_2_) (Poon et al. 2014).

The membranolytic activity of ACPs depends on the intrinsic characteristics of the peptide as well as the properties of the target membrane (Mulder et al. 2013). Also, the selectivity of some ACPs against cancer cells depends on the net negative charge of the membrane (Gaspar et al. 2013). Anionic molecules (phosphatidylserines, glycoproteins, glycosaminoglycans, heparan sulfate, *O*-glycosylated mucins, and sialylated gangliosides) confer a net negative charge on the membranes of cancer cells, in contrast to the typically zwitterionic membranes of normal cells (Giuliani et al. 2007; Hoskin and Ramamoorthy 2008; Raz et al. 1980; Schweizer 2009; Utsugi et al. 1991). The mode of action may involve electrostatic interactions between cationic peptides and the anionic components of cancer cell membranes (Kim et al. 2013). The same “carpet-like” and “barrel-stave” models that explain the interaction between AMPs and bacterial membranes can therefore also be invoked to describe interactions with cancer cells (Oren and Shai 1998; Pouny and Shai 1992; Schweizer 2009). Additional membranolytic events involve the permeabilization and swelling of mitochondria, followed by the release of cytochrome c and the induction of apoptosis (Mai et al. 2001).

Although the rapid killing of cells by ACPs may indicate the prevalence of a non-receptor-mediated mode of action, some non-membranolytic activities have also been described (Sharma 1992; Wachinger et al. 1998; Winder et al. 1998). These include the inhibition of angiogenesis, which is essential for the formation of tumor-associated vasculature (Schweizer 2009). Peptides can block the function of receptors expressed on angiogenic endothelial cells and thus perturb the formation of the tumor-associated vasculature (Arap et al. 1998; Lee et al. 2011; Mader and Hoskin 2006; Rosca et al. 2011; Schweizer 2009). The primary objective of antiangiogenic therapy is to normalize the tumor vasculature instead of reducing the density of tumor blood vessels (Shang et al. 2012). The development of therapeutic molecules which, individually or in combination with other reagents, target several aspects of angiogenesis might prove fruitful for cancer treatment in the future (Rosca et al. 2011).

## Impact of insect AMPs on cancer

Insects comprise ∼55 % of total biodiversity and ∼85 % of animal biodiversity (Chernysh et al. 2002) and therefore provide a large potential source of ACPs. Only a few AMPs have been identified as ACPs based on in vivo testing, although others have been tested against tumor cell lines (Table 1). Insect-derived ACPs have not been tested directly against skin cancer cells, but as stated above, Rodrigues et al. (2008) successfully showed that the spider peptide gomesin was effective against subcutaneous murine B16F10-Nex2 melanoma cells when administered topically. The efficacy of insect ACPs against other types of cancer cells provides evidence that they should also be active against skin cancer. For example, cecropin B from *Hyalophora cecropia* increased the survival of mice bearing ascitic murine colon adenocarcinoma cells (Moore et al. 1994). Alloferon 1 isolated from bacteria-challenged larvae of the blow fly *Calliphora vicina* was able to stimulate NK cell activity and interferon (IFN) synthesis in animal and human models and could enhance antiviral and antitumor activity in mice (Chernysh et al. 2002). The harmoniasin analog HaA4 was found to be cytotoxic toward human leukemia cell lines such as U937 and Jurkat cells by inducing both caspase-dependent apoptosis and necrosis (Kim et al. 2013). D-peptides A, B, C, and D, designed and synthesized based on the sequences of 43-mer defensins from two beetles, were able to inhibit the growth of several cancer cell lines with different levels of efficacy, and D-peptide B showed the most selective activity against the mouse myeloma cell line P3-X63-Ag8.653. Flow cytometry and scanning electron microscopy revealed that this peptide disrupts myeloma membrane construction but has no effect against normal leukocytes. In addition, combinations of D-peptide B and dexamethasone showed synergistic activity against a mouse myeloma cell line (Iwasaki et al. 2009). These peptides are therefore promising candidates for novel anticancer drugs.

**Table 1:** Insect antimicrobial peptides with activity against cancer cells

ACP	Origin	Sequence	Cancer cell type	Anticancer mechanism	Hemolytic activity	Reference
HaA4	*Harmonia axyridis*	IGGYCSWLRL	U937 and Jurkat	Necrosis and caspase-dependent apoptosis	No	Kim et al. (2013))
D-peptide B	Synthetic peptide	RLRLRIGRR	P3-X63-Ag8.65	Depolarization, membrane disruption	3 % at 640 μM	Iwasaki et al. (2009))
CopA3	*Copris tripartitus*	LLCIALRKK	Human gastric cancer cells	Apoptosis and necrosis	ND	Lee et al. (2015))
			Human leukemia cells	Caspase-independent, AIF-mediated apoptosis	ND	Kang et al. (2012))
Lasioglossins (LL-III/1)	*Macropis fulvipes* *Macropis fulvipes* *Macropis fulvipes* *Macropis fulvipes*	VNWKKILAKIIKVVK	Hela S3, CEM, HUVEC, IEC, and SW	Permeabilization of the cell membrane	No	Slaninova et al. (2012))
Halictines (HAL-1/18)		GMWSKILKHLIR	HeLa S3 and CEM			
Macropin 1		GFKMALKLLKKVL	Hela S3 and CEM			
Macropin 2		GTGLPMSERRKIMLMMR	CEM			
Alloferon 1	*Calliphora vicina*	HGVSGHGQHGVHG	P388	Stimulation of NK cell activity and IFN synthesis	ND	Chernysh et al. (2002))
Alloferon 2		GVSGHGQHGVHG				
Melittin	*Apis mellifera*	GIGAVLKVLTTGLPALISWIKRKRQQG	Human hepatocellular carcinoma	Influx of Ca^2+^/carpet mechanism/toroidal pore	ND	Tosteson et al. (1985); Wang et al. (2009))
				Suppression of cathepsin S activation, components of the VEGF, MAPK1, and ERK		Zhang et al. (2016))
Cecropin A	*Hyalophora cecropia*	KWKLFKKIEKVGQNIRDGIIKAGPAVAVVGQATQIAK	HL-60	Caspase-independent, ROS-mediated apoptosis	ND	Ceron et al. (2010))
Cecropin B	*Antheraea pernyi*	KWKIFKKIEKVGRNIRNGIIKAGPAVAVLGEAKAL	LS-174T	ND	ND	Zhang et al. (2003))
Cecropin	*Musca domestica*	GWLKKIGKKIERVGQHTRDATIQTIGVAQQAANVAATLK	BEL-7402	Apoptosis-inducing properties	ND	Jin et al. (2010))
Cecropin XJ	*Bombyx mori*	RWKIFKKIEKMGRNIRDGIVKAGPAIEVLGSAKAIGK	HeLa, Hep2, BGC823	Concentration-dependent manner, cytoskeleton disruption, disruption of mitochondrial membrane potential	No	Xia et al. (2013), 2016); Wu et al. (2015))
Mastoparan	*Vespula lewisii*	INLKALAALAKKIL	B16F10-Nex2 melanoma cells	Mitochondrial apoptosis pathway	ND	de Azevedo et al. (2015))
Halictine 1	*Halictus sexcinctus*	GMWSKILGHLIR	Potency to kill several cancer cells (author’s unpublished results).	ND	No	Monincova et al. (2010))
Lasioglossin LL-I	*Lasioglossum laticeps*	VNWKKVLGKIIKVAK	PC12	ND	LC_50_ >200	Čeřovský et al. (2009))
Lasioglossin LL-II		VNWKKILGKIIKVAK				
Lasioglossin LL-III		VNWKKILGKIIKVVK	L1210, CCRF-CEM T, HL-60, HeLa S3, PC12, SW480			
Polybia-MPI	*Polybia paulista*	IDWKKLLDAAKQIL	EJ, PC-3, HEPG-2		30 % at 100 μM	Zhang et al. (2010))

*AIF* apoptosis-inducing factor, *BEL-7402* human hepatocellular carcinoma cell line, *BGC823 cells* human gastric cancer, *CCRF-CEM T* human lymphoblastic leukemia, *CEM* a cell line derived from human T cells, *ERK* extracellular signal-regulated kinase, *HeLa* human cervical cancer, *HeLa S3* a clonal derivative of the parent HeLa line, cervix tissue, *Hep2* human laryngeal cancer, *HL-60* human promyelocytic leukemia cells, *HUVEC* human umbilical vein endothelial cells, *IEC* intestinal epithelial cells, *L1210* mouse lymphocytic leukemia, *LS-174T* human colon adenocarcinoma cell line, *PC12* pheochromocytoma of the rat adrenal medulla, *P3-X63-Ag8.65* mouse myeloma cell line, *P388* leukemia cells, *SW* human colon carcinoma cells, *SW480* human colon adenocarcinoma, *U937 and Jurkat* human leukemia cell, *VEGF* vascular endothelial growth factor

## AMPs as prophylactic anticancer ingredients in cosmetics

As stated above, skin cancer can often be cured by surgery and/or chemotherapy following an early diagnosis, although both approaches carry a moderate risk of recurrence (Guerra-Rosas and Álvarez-Borrego 2015). However, skin cancer is also one of the easiest diseases to prevent, because exposure to UV radiation can be limited not only by wearing appropriate clothing and staying indoors, but also by applying barrier creams that block the most dangerous wavelengths of UV radiation. The success of such approaches depends on the compliance of an informed at-risk population, because even the strongest barrier creams have a limited effective duration of activity. Therefore, the development of novel products that provide additional protection would reduce the incidence of skin cancer even further. Endogenous AMPs are produced in the human skin, so additional peptides with broader properties (including anticancer activity) could be used not only as a therapeutic intervention but also as a prophylactic measure to counteract cancer cell development by including the peptides in barrier creams, ointments, functionalized wound dressings, and cosmetics.

## Challenges of insect AMPs

Although AMPs from insects and other sources could be developed into new products for the prevention and treatment of cancer, one challenge is the high cost of synthesis because many AMPs are long and contain disulfide bridges. However, the anticancer activity of AMPs is likely to resolve to certain motifs, and if these motifs can be identified, they could be produced in the context of a smaller artificial peptide. Furthermore, different AMPs can complement each other via potentiating interactions including synergy (Rahnamaeian et al. 2016; Bolouri Moghaddam et al. 2016). Therefore, hybrid peptides containing functional motifs from different AMPs could achieve greater therapeutic efficacy. Another concern is the hemolytic activity of some AMPs, and peptide engineering would be necessary to maximize their anticancer activity while minimizing hemolysis, e.g., by increasing the positive charge and hydrophobicity as well as changes in the spatial configuration of particular amino acids (Rahnamaeian and Vilcinskas 2015). The pH sensitivity of cationic AMPs also affects their activity, so the pH and ionic strength of the carrier matrix must be optimized to achieve the greatest efficacy.

## Concluding remarks

AMPs have drawn the attention of the pharmaceutical industry because they represent a promising next generation of drugs with different modes of action compared to current antitumor agents. The latter tend to have severe side effects and encourage the development of resistant cell populations. AMPs offer a number of advantages over contemporary drugs and anticancer vaccines, including their potent activity at low concentrations, their high specificity (hence low toxicity toward normal cells), and the ability to produce them as cost-effective synthetic or recombinant peptides, particularly short AMPs without disulfide bonds. AMPs provide a promising source of new drugs for the prevention and treatment of skin cancer because they are highly suitable for topical application and can be formulated as creams and ointments, which are suitable for self-administration or for parents to apply to their children.
